# The impact of early social support on subsequent health recovery after a major disaster: A longitudinal analysis

**DOI:** 10.1016/j.ssmph.2021.100779

**Published:** 2021-03-26

**Authors:** Bonnie Khanh Ha Bui, Philip Anglewicz, Mark J. VanLandingham

**Affiliations:** a Tulane University School of Public Health and Tropical Medicine, USA; b Johns Hopkins Bloomberg School of Public Health, USA

**Keywords:** *Social support*, *Disaster recovery*, *Immigration*

## Abstract

Social support may facilitate disaster recovery. Prior analyses are hampered by the limits of cross-sectional approaches. We use longitudinal data from the KATIVA-NOLA survey to explore whether social support soon after Hurricane Katrina facilitated recovery of health status for a representative sample of 82 Vietnamese New Orleanians. Health and social support were assessed just before Hurricane Katrina (2005), soon afterwards (2006, 2007), and at longer durations post-disaster (2010, 2018). We use random effects regression to examine how social support measured in 2006 influences mental and physical health measured in 2006, 2007, 2010, and 2018. Social support soon after Katrina was positively associated with physical health and mental health years later in 2010, even after controlling for potential confounders such as Katrina-related housing damage and pre-Katrina health and support and modeling an interaction between year and social support in 2006. Other immigrants who are highly impacted by a major disaster could benefit from programs that seek to rapidly reconstruct systems of social support.

## Highlights

• We use longitudinal data to explore if social support soon after Hurricane Katrina facilitated recovery for Vietnamese.
• We use random effects regression to examine how social support measured in 2006 influences health in the following years.
• Social support soon after Katrina was positively associated with health, even after controlling for potential confounders.
• Immigrants could benefit from programs seeking to rapidly reconstruct social support systems after a major disaster.
• Early social support may facilitate disaster recovery.

## Background

Social support is strongly implicated in health and well-being (House et al., 1988; Umberson & Montez, 2010). In ordinary times, helpful social relations can facilitate mental health by providing sounding boards for problem solving, emotional sustenance, and active coping assistance (Thoits, 2011). Functional support, both instrumental and informational, prevents or protects against mental distress (Lin et al., 1999), particularly for adults less than 60 years of age (Peek & Lin, 1999). Social contacts also facilitate physical health and well-being by spreading important information about potential health hazards and opportunities for improving health that an individual may have otherwise been unaware of. These functions of advice, comfort, and information sharing are especially important within immigrant communities, whose residents are often isolated from mainstream populations by language, culture, and geography.

In extraordinary times, for example during the aftermath of a major disruption caused by a natural or manmade disaster, social support may play an important role in determining who recovers well and who does not (Abramson et al., 2010). In other words, social relations may be a central feature of resilience (Norris et al., 2008). For immigrants, strong social support might well provide important sources of advice and comfort as families navigate the post-disaster landscape, and might well mitigate some of the negative effects of isolation, *e.g.*, by sharing information about how to navigate unfamiliar and complex government and insurance bureaucracies.

Disasters have a profound impact on physical (Galea, 2007; Van Den Berg et al., 2005) and mental health (Galea, 2007; Galea et al., 2005; Goldmann & Galea, 2014; Neria et al., 2008). Some of the adverse impacts from disasters are due to the unexpected nature and large scale of destruction that disrupt existing social networks, harm community resources, and cause injury to individuals (Goldmann & Galea, 2014). Social support may facilitate recovery from a disaster, but this relationship has seldom been tested with longitudinal data, nor has it been explored longitudinally for immigrant populations.

Immigrants experience differential exposure and vulnerability to disaster-related trauma compared to native-born (Perilla et al., 2002), and these difficulties can be especially pronounced for immigrants with limited fluency in the local language spoken in their new destination country. Immigrants are particularly vulnerable to disasters for a number of reasons. First, immigrants often establish enclaves in urban areas that are prone to flooding, fire, and government neglect, since prices tend to be cheaper in these areas, and many immigrants arrive with modest economic resources (Blaikie et al., 2014). Second, some immigrants, and refugees in particular, often bring with them physical and mental vulnerabilities that are related to the calamity that displaced them in the first place (Sutker et al., 2002). Third, due to difficulties with the local language and customs, newly-arrived immigrants may find it more difficult to access government and non-government resources than do citizens with a longer history in the community (Wisner, 1998). For immigrants, social support from non-official channels may be crucial for recovery from a disaster.

Understanding how social support affects post-disaster health is essential for helping communities and individuals recover from these calamities. The flooding of New Orleans after Hurricane Katrina revealed the vulnerabilities in preparedness and difficulties in recovery for ethnic minority communities (Chen et al., 2007). Immigrant communities are often physically, socially, and politically isolated from mainstream populations and thus face special circumstances that may shape the role that social networks play in their post-disaster health trajectories. We explore the relations between social support and post-disaster health within New Orleans’ largest immigrant population, the Vietnamese American community, which was heavily flooded by Hurricane Katrina during August 2005.

Social support has been linked to both mental health (Umberson & Montez, 2010) and physical health (Uchino, 2006). For instance, among cancer survivors, lower social support is linked to negative mental and physical health outcomes (Hughes et al., 2014). Social support may also enhance disaster recovery, as suggested by evidence from Hunan, China, where recovery from a major flood 13–14 years later was associated with higher levels of social support, which was assessed at this same long-term follow-up (Dai et al., 2016). While intriguing, this important study of long-term recovery leaves unanswered the question of whether social support led to subsequent recovery, or whether both the level of social support and the degree of long-term recovery were both driven by some other factors that may have been present before the disaster occurred. Furthermore, recall is potentially unreliable, especially if the respondent is asked to reach back many years (Frankenberg et al., 2020). To answer these questions, a longitudinal approach with pre-disaster measures and an assessment of social support early in the recovery process seem essential.

Some of the earlier research has suggested that the quality of social support is related to physical and mental health while social network size is not (Vandervoort, 1999), while other research has suggested that small networks are associated with particular adverse health outcomes, like mortality (Berkman & Syme, 1979). Some of this inconsistency could be a result of looking at ties more broadly instead of focusing on ties that were deemed to have substantial impact—that is, ties that are deemed to be helpful. Also, much of this existing research is hampered by an inability to tease out the potentially confounding influences of early social support on subsequent assessments that are of more central interest. In this study, we control for the presence of helpful ties before Katrina in order to understand how social support as assessed soon after this disaster might influence subsequent physical and mental health within the Vietnamese American immigrant community in New Orleans.

Conceptually, the relationships among social support, disasters, and health outcomes are complex. Two major functions of social networks are to facilitate the flow of information and to provide sources of support during a stressful situation. Disasters can dismantle existing social support networks and disrupt the flow of important information. Disasters also disperse communities, and in doing so remove usual sources of comfort and consolation (Kaniasty & Norris, 1995). These disruptions may well be especially consequential for health and wellbeing within immigrant enclaves, since social ties are typically dense within the enclave but sparse outside of it (Zhou & Bankston, 1999). Vietnamese immigrants are an especially segregated immigrant group (Hall, 2013), leaving many of them linguistically and culturally isolated from mainstream society as well (Ramakrishnan & Ahmad, 2014; Siegel et al., 2001).

Finally, as noted in our review above of the important study in Hunan, China, the timing of events is of central importance in a conceptual framework linking social support, disasters, and recovery of health status. Cross-sectional studies often find a relationship between social support and health outcomes after a major disaster but lack the leverage that a longitudinal approach provides to sequence such events in time. There is a need for more studies that take a life-span perspective in linking social support and physical health (Uchino, 2009). A longitudinal framework makes it possible to discern whether social support soon after a disaster can influence health recovery trajectories, controlling for circumstances that existed prior to the disaster of interest as well as important factors related to the disaster itself. Other studies that assess how circumstances of the pre-disaster and early recovery periods influence survival and later well-being illustrate the critical importance of a longitudinal approach with data that include pre-disaster measures, an assessment of the situation soon after the disaster occurs, and long-term follow-up (Frankenberg et al., 2020; Murdock et al., 2018).

**Fig. 1:**
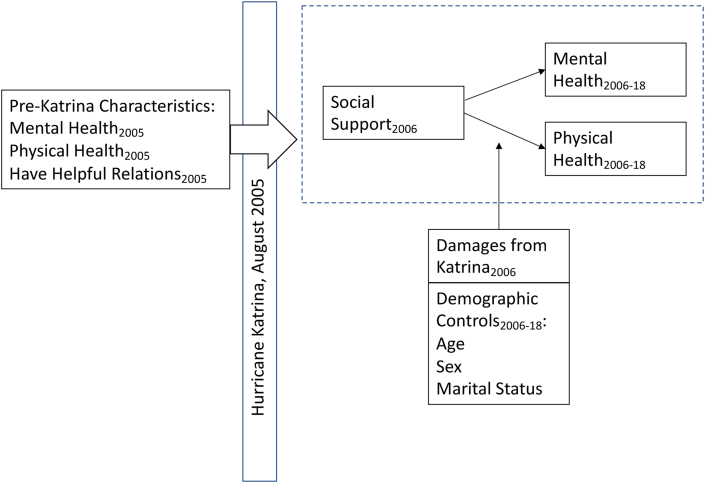
A conceptual framework: Early post-disaster social support and longer-term physical and mental health outcomes.

We focus on how social support soon after Katrina influences mental and physical health in later years. Our conceptual framework is illustrated in Fig. 1. Our central hypothesis is that helpful social relations soon after a disaster will have a positive relationship with subsequent mental and physical health outcomes, even after controlling for a wide range of pre-disaster and post-disaster characteristics that might confound such a relationship. We test this hypothesis using a longitudinal framework. We use a measure of social support and social embeddedness in 2006 shortly after Katrina, while controlling for prior health status and having helpful ties assessed just prior to Katrina, to determine whether social support right after a disaster matters for long-term physical and mental health. If this hypothesis is confirmed, it would have important and actionable public health implications that could facilitate the recovery of especially vulnerable populations after a major disaster.

## Data and methods

### Methods

We use rarely-available longitudinal panel data from a representative sample of Vietnamese Americans living in the greater New Orleans area (KATIVA NOLA 2005–2018), which includes measures of health and social support just before Hurricane Katrina (2005), soon afterwards (2006, 2007), and at longer durations after the disaster (2010, 2018). With these panel data, we examine how social support assessed soon after Hurricane Katrina (2006) influences mental and physical health measured in later years (2007, 2010, and 2018), all while controlling for pre-Katrina physical and mental health and support (measured by the number of helpful relations).

The KATIVA-NOLA study has several measures of both health and social support. Our measure of physical health is the SF-36/SF-12 physical health summary score; our measure of mental health is the SF-36/SF-12 mental health summary score. Our key independent variable is social support, measured by the Louisville Social Support and Social Embeddedness Scale (LSSS), measured in 2006. We also include whether the respondent had any helpful relations in 2005, derived from the Social Relations Scale, as a control. This was done to ensure that any observed effects on physical and mental health are due to social support soon after Katrina and not due to having helpful relations before Katrina, which could explain better recovery outcomes. We also control for Katrina-related losses and for pre-Katrina (physical and mental) health and other demographic characteristics (age, gender, and marital status). Our pre-Katrina measures were taken before Hurricane Katrina occurred.

### Data

The first wave of this panel survey took place during the summer of 2005, just weeks before Hurricane Katrina struck and flooded the Gulf Coast. This sample in the first wave consists of 125 first-generation Vietnamese immigrants living in New Orleans. Respondents were randomly selected from a recently updated register of the entire population of Vietnamese Americans living in New Orleans. Because the original (pre-Katrina) study was of immigrant adaptation to their new lives in the U.S., eligible individuals were age 20–54 at the time of the first wave, were born in Vietnam, arrived in the United States between 1975 and 1990, and were older than 5 years of age when they arrived. The response rate was 74%. Our analyses use the follow-up waves in 2006, 2007, 2010, and 2018, but use measures from the first wave to account for pre-Katrina characteristics. The follow-up wave in 2006 consists of 82 respondents, which is the analytic sample of the unbalanced panel data models using random effects.

### Measures

Standard subscales based on the Medical Outcomes Study (MOS) Short Form-36 (SF-36) (2005, 2006, 2007) and Short Form-12 (SF-12) (2010, 2019) health assessment instruments were employed to assess the mental health and physical health status of our respondents. The MOS SF-36/12 include items that form subscales for the following eight indicators: physical functioning (PF), role physical (RP), bodily pain (BP), general health (GH), vitality (VT), social functioning (SF), role emotional (RE), and mental health (MH). These subscales are combined to form composite physical and mental health component scores that range from 0 (lowest functioning) to 100 (highest functioning). The mental component summary (MCS) scores and physical component summary (PCS) scores for the SF-36 and SF-12 were calculated using the formulas by Ware et al. (Ware et al, 1993, 1998). The SF-12 and SF-36 have been tested in studies examining their consistency over time in longitudinal studies, and they have found to be near identical (Jenkinson et al., 1997), and so both are used in this study. Our team worked closely with the developers of the instruments to adapt and test them for Vietnamese-speaking populations (Fu & VanLandingham, 2012).

For our primary measure of social support, we used the Louisville Social Support and Social Embeddedness Scale (LSSS), which consists of 13 items that measure the extent and closeness of the respondent's social network and degree of satisfaction with support provided by that social network. Respondents are asked questions about how often they see friends, family, or neighbors; participate in clubs; and how much help they can expect to receive from friends, family, or neighbors during an emergency. All responses are categorical, and the number of responses for each question ranges from 3 to 5, so it was necessary to standardize all the items first before creating an averaged scale. The scale demonstrated good internal consistency (Cronbach's α=.81). Higher scores indicate higher social support.

We included baseline measures for physical health, mental health, and whether the respondent had helpful relations in our model to control for pre-Katrina characteristics that would impact physical and mental health outcomes. We used PCS and MCS, derived from the SF-36 in 2005. Our indicator variable for whether the respondent had helpful relations was derived from the Social Relations Scale (SRS), which includes questions about whether a respondent discusses home and family with another person, the relationship with that person, the degree of helpfulness of the discussion with that person, and reciprocal relations with that person in discussing home and family. With the SRS, we derived a measure for the number of alters that the respondent reported was helpful (determined by values 5–7 on a 7-point scale). We then created an indicator variable, with any respondent having at least one helpful tie as “1,” and respondents with no helpful ties as “0.” We also derived other measures (*i.e.*, number of named relations, reciprocity, or the proportion of people with whom you talk who also talk to you about home and family), but these measures were excluded from the study because of the high number of missing values and/or because they were not correlated with our health outcome measures.

We controlled for damages from Katrina in 2006. We constructed a summed scale from 7 items asking about the extent of damages to the following: house and property, business, furniture, appliances, sentimental possessions, external structure of house, and motor vehicles. Responses for these items were as follows: “1” for “none or minimal damage,” “2” for “slight damage,” “3” for “moderate damage,” “4” for “severe damage,” and “5” for total destruction. The scale demonstrated good internal consistency (Cronbach's α=.88). Demographic covariates included in our models are gender, age, and marital status.

### Analysis

We use random effects models to examine the associations among social support, physical health, and mental health while controlling for pre-Katrina characteristics, demographics, and Katrina-related damages. The goal of these analyses is to determine whether social support early during the recovery period might affect mental and physical health and if this association differs across time. We model this by adding interaction terms for social support in 2006 with each indicator for each subsequent wave to test whether the effect that early social support has on physical and mental health outcomes differ across years while also controlling for the recovery that happens with time. All analyses were done using Stata 15.1.

## Results

Table 1 presents the mean, standard deviation, range, and number of observations for each variable used in the unbalanced panel models. The mean age of the sample 48 years. About 83% reported that they were married or cohabiting. 35% of the sample was female. For this generation, this gender distribution is representative of Vietnamese Americans living in the New Orleans metro area, as refugees leaving Vietnam were mostly male (See Goodkind, 1997 for more details on the demographic consequences of this gender imbalance.). The physical health component score (PCS) was lower after Katrina compared to pre-Katrina levels (50.2 compared to 53.9 before Katrina). The mental health component score (MCS) was about the same pre- and post-Katrina. In 2005, 92% reported that they had at least one helpful tie.

**Table 1:** Descriptive statistics of variables used in unbalanced panel.

Variables	Mean or %	SD	Min	Max	Obs.
*SF Component Scores*					
Physical Health (PCS)	50.186	(8.465)	19.654	67.088	337
Mental Health (MCS)	48.441	(10.601)	13.649	68.700	337
*Social Support*					
Louisville Social Support and Social Embeddedness Scale (*z*-transformed) (LSSSE 2006)	0.013	(.546)	−1.471	1.010	280
*Demographic and Other Covariates*					
Married or Cohabiting	82.62%				328
Age	47.726	(6.324)	28	65	329
Female	35.35%				331
Damages from Katrina Scale (2006)	23.157	(7.574)	6	35	280
*Pre-Katrina controls in 2005*					
Have Helpful Relations (2005)	92.28%				324
PCS (2005)	53.871	(6.522)	32.908	62.815	324
MCS (2005)	47.923	(5.330)	28.899	57.717	324

*Note:* Summary statistics reported are for an unbalanced panel across four waves of data (2006, 2007, 2010, and 2018). Pre-Katrina characteristics were measured in the initial 2005 wave (not included in the random effects models); these observations are repeated through the subsequent waves of a longitudinal panel (2006, 2007, 2010, 2018) to measure the time-invariant impact of important pre-hurricane characteristics on physical and mental health. Social Support and Damages from Katrina were measured in 2006 to capture the time-invariant impact of early social support.

Next, we present the results from our random effects models to examine predictors of physical and mental health in Table 2, Table 3, respectively. In both tables, Model 1 includes demographic covariates, social support and damages from Katrina in 2006, and indicators for each year. In Model 2, pre-Katrina characteristics were added to the model to ensure that whatever associations were seen between social support and PCS/MCS were not a result of having helpful relations or having good physical and mental health to begin with. Model 3 adds in interactions of social support with each wave (2007, 2010, and 2018).

**Table 2:** Random effects models for the effects of social support and other covariates on physical health: 2006–2018.

Variables	Model 1			Model 2			Model 3		
	Coef.	SE	Sig.	Coef.	SE	Sig.	Coef.	SE	Sig.
*Social Support*									
Louisville Social Support and Social Embeddedness Scale (LSSSE 2006)	3.360	(1.287)	**	3.653	(1.247)	**	3.572	(1.610)	*
*Demographic and Other Covariates*									
Married or Cohabiting	−0.760	(1.539)		−0.989	(1.513)		−0.983	(1.533)	
Age	−0.430	(.139)	**	−0.500	(.139)	***	−0.506	(.137)	***
Female	−0.643	(1.479)		0.303	(1.441)		−0.187	(1.421)	
Damages from Katrina (2006)	−0.160	(.092)	†	−0.127	(.089)		−0.130	(.088)	
*Pre-Katrina controls in 2005*									
Have Helpful Relations (2005)				6.809	(2.615)	**	6.844	(2.576)	**
PCS (2005)				0.108	(.109)		0.109	(.107)	
*Year*									
2007	2.975	(1.010)	**	3.037	(1.007)	**	3.053	(1.016)	**
2010	−1.946	(1.217)		−1.678	(1.213)		−1.641	(1.218)	
2018	2.473	(1.971)		3.182	(1.959)		3.452	(1.958)	†
*Interactions of Social Support in 2006 and Year*									
LSSSE2006 × 2007							0.454	(1.808)	
LSSSE2006 × 2010							0.934	(1.927)	
LSSSE2006 × 2018							−1.778	(2.202)	
Intercept	74.088	(6.486)	***	64.370	(9.172)	***	64.622	(9.040)	***
*N* (observations)	274			274			274		
(Overall) *R*^2^	0.2180			0.2622			0.2627		
*N* (individuals)	82			82			82		
*R*^2^ within	0.1709			0.1696			0.1794		

*Note:* Models 2 and 3 add in pre-Katrina control variables from 2005 to the random effects models. Pre-Katrina characteristics are repeated through the longitudinal panel (2006–2018) to measure the time-invariant impact of important pre-hurricane characteristics on physical and mental health. LSSS 2006 and damage from Katrina in 2006 were also repeated through the waves to measure the time-invariant impact of social support in 2006 on physical and mental health while controlling for damages from Katrina in 2006.

†*p* < .10, *p < .05, **p < .01, ***p < .001 (two-tailed tests).

**Table 3:** Random effects models for the effects of social support and other covariates on mental health: 2006–2018.

Variables	Model 1			Model 2			Model 3		
	Coef.	SE	Sig.	Coef.	SE	Sig.	Coef.	SE	Sig.
*Social Support*									
Louisville Social Support and Social Embeddedness Scale (LSSSE 2006)	2.751	(1.399)	*	3.237	(1.401)	*	1.212	(1.985)	
*Demographic and Other Covariates*									
Married or Cohabiting	4.998	(1.895)	**	4.801	(1.876)	*	4.068	(1.880)	*
Age	−0.269	(.153)	†	−0.351	(.154)	*	−0.358	(.152)	*
Female	−0.305	(1.614)		−0.268	(1.583)		−0.130	(1.573)	
Damages from Katrina (2006)	−0.221	(.100)	*	−0.210	(.099)	*	−0.212	(.099)	*
*Pre-Katrina controls in 2005*									
Have Helpful Relations (2005)				6.811	(2.867)	*	6.890	(2.844)	*
MCS (2005)				−0.087	(.144)		−0.075	(.143)	
*Year*									
2007	4.209	(1.385)	**	4.269	(1.383)	**	4.280	(1.363)	**
2010	7.239	(1.598)	***	7.555	(1.596)	***	7.613	(1.575)	***
2018	11.426	(2.383)	***	12.199	(2.375)	***	12.508	(2.360)	***
*Interactions of Social Support in 2006 and Year*									
LSSSE2006 × 2007							1.410	(2.440)	
LSSSE2006 × 2010							7.199	(2.592)	**
LSSSE2006 × 2018							−0.421	(2.946)	
Intercept	56.597	(7.152)	***	58.061	(10.167)	***	58.315	(10.086)	***
*N* (observations)	274			274		274			
(Overall) *R*^2^	0.1886			0.2183		0.2381			
*N* (individuals)	82			82		82			
*R*^2^ within	0.1353			0.1329		0.1811			

*Note:* Models 2 and 3 add in pre-Katrina control variables from 2005 to the random effects models. Pre-Katrina characteristics are repeated through the longitudinal panel (2006–2018) to measure the time-invariant impact of important pre-hurricane characteristics on physical and mental health. LSSS 2006 and damage from Katrina in 2006 were also repeated through the waves to measure the time-invariant impact of social support in 2006 on physical and mental health while controlling for damages from Katrina in 2006.

†*p* < .10, *p < .05, **p < .01, ***p < .001 (two-tailed tests).

In Model 1 of Table 2, we find that social support in 2006 (*b* = 3.360, *p* < .01) was positively associated with physical health. Age (*b* = −0.430, *p* < .01) was negatively associated physical health. There also appeared to be a recovery effect in 2007 independent of the other measured covariates (*b* = 2.975, *p* < .01), and this recovery effect remained after adding in pre-Katrina measures (*b* = 3.037, *p* < .01) and the interactions between 2006 social support and each year indicator (*b* = 3.053, *p* < .01). Adding in pre-Katrina controls for having helpful ties and physical health in Model 2 did not result in meaningful changes to the association of social support in 2006 and physical health. Having helpful ties pre-Katrina was positively associated with physical health (*b* = 6.809, *p* < .01). Social support (*b* = 3.653, *p* < .01) and age (*b* = −0.500, *p* < .001) remain associated with physical health. Adding the interaction of social support with each year in Model 3 did not alter any associations demonstrated by Models 1 and 2.

Table 3 presents similar models to that shown in Table 2, for mental health as the outcome variable. Similar to physical health, age was negatively associated with mental health, and having helpful relations pre-Katrina was positively associated with mental health. Additional findings were that cohabiting or being married was positively associated with mental health, and having damages from Katrina was negatively associated with mental health. The main effects for year show that each passing year resulted in increases in mental health (the coefficients increased each year in magnitude, from about 4 in 2007 to 7 in 2010 and about 12 in 2018). These findings hold across all three models. However, in Model 3 when the interactions were added to the model, social support in 2006 was no longer positively associated with mental health. Instead, we see that the interaction of 2006 social support and the year 2010 is positive (*b* = 7.199, *p* < .01). This shows that social support in 2006 was positively associated with better mental health in 2010.

We also modeled a time-varying measure of social support up to the year 2010, as we did not include the measure in our 2018 wave. The results for those models are not presented in this paper. However, the time-varying measure of social support was not significantly associated with physical or mental health, whereas our models show that 2006 social support does matter for physical and mental health. This lends support to our hypothesis that the timing of social support matters.

## Discussion

Our central hypothesis is confirmed: early social support after a disaster is indeed correlated with physical and mental health. In this study of how the Vietnamese American immigrant community fared after Hurricane Katrina, we found that social support in 2006 was positively associated with both physical and mental health, even while accounting for pre-Katrina features such as physical health, mental health, and having helpful relations; and key post-Katrina features such as housing damage.

Our longitudinal approach makes possible several contributions to the existing literature on social support and post-disaster recovery of health status. First, this longitudinal approach allows us to confirm that early social support post-disaster is associated with health outcomes across time. Second, this approach allows us to control for the potentially confounding influence of pre-disaster health and network size and disaster-related damages on the relationship between early post-disaster social support and long-term health outcomes.

We acknowledge several limitations of the study. First, the sample sizes for this study are small, which limits our ability to detect other findings of potential significance and importance. Second, because of the lack of a robust baseline measure of social support in 2005, we had to proxy this measure by using a measure of helpful relations in 2005.

Our study's approach and findings have important methodological, substantive, and policy implications. Methodologically, our study illustrates the importance of pre-disaster data, an assessment of the immediate post-disaster situation, long-term follow-up, and a longitudinal approach for research that seeks to understand how features in play soon after a disaster influence health outcomes both immediately and further down the road. Substantively, within an immigrant community highly impacted by Hurricane Katrina, strong social support during the early phases of post-disaster recovery is positively associated with subsequent high levels of physical and mental health. These central findings have important policy implications. Other immigrant communities share important characteristics with our community of interest here (Cuervo et al., 2017), including dense and helpful social ties within the enclave and social, cultural, and linguistic isolation from broader society. Other immigrant communities also share similar vulnerabilities when a major disaster results in the dispersal of these usual sources of social support (Nguyen & Salvesen, 2014). Our results strongly suggest that programs that help to re-establish disrupted social ties soon after a disaster could facilitate long-term recovery in mental and physical health among members of these especially vulnerable communities.

## Ethical statement

Ethical approval for this study was obtained from the Tulane University Human Research Protection Office.

## Funder

Our research is funded by the Eunice Kennedy Shriver National Institute for Child and Human Development, 10.13039/100000002National Institutes of Health (R03HD042003 VanLandingham PI; R21HD057609 VanLandingham PI; and P01HD082032 VanLandingham Contact PI).

## CRediT authorship contribution statement

**Bonnie Khanh Ha Bui:** Conceptualization, Methodology, Software, Formal analysis, Writing – original draft, Writing – review & editing, Visualization. **Philip Anglewicz:** Data curation, Methodology, Writing – review & editing. **Mark J. VanLandingham:** Conceptualization, Methodology, Writing – review & editing, Funding acquisition.

## Declaration of competing interest

None.
